# The biochemical recurrence-free rate in patients who underwent prostate low-dose-rate brachytherapy, using two different definitions

**DOI:** 10.1186/1748-717X-9-107

**Published:** 2014-05-06

**Authors:** Nobumichi Tanaka, Isao Asakawa, Emiko Katayama, Akihide Hirayama, Masatoshi Hasegawa, Noboru Konishi, Kiyohide Fujimoto

**Affiliations:** 1 Department of Urology, Nara Medical University, 840 Shijo-cho, Kashihara, Nara 634-8522, Japan; 2 Department of Radiation Oncology, Nara Medical University, Kashihara, Nara, Japan; 3 Department of Pathology, Nara Medical University, Kashihara, Nara, Japan; 4 Department of Urology, Nara Hospital Kinki University Faculty of Medicine, Ikoma, Nara, Japan

**Keywords:** Prostate cancer, LDR-brachytherapy, Biochemical recurrence-free rate, BED

## Abstract

**Background:**

To assess the biochemical recurrence (BCR)-free rate in patients who underwent prostate low-dose-rate brachytherapy (LDR-brachytherapy), using two different definitions (Phoenix definition and PSA ≥ 0.2 ng/mL).

**Methods:**

Two hundreds and three patients who were clinically diagnosed with localized prostate cancer (cT1c-2cN0M0) and underwent LDR-brachytherapy between July 2004 and September 2008 were enrolled. The median follow-up period was 72 months. We evaluated the BCR-free rate using the Phoenix definition and the PSA cut-off value of 0.2 ng/mL, as in the definition for radical prostatectomy. To evaluate an independent variable that can predict BCR, Cox’s proportional hazard regression analysis was carried out.

**Results:**

The BCR-free rate in patients using the Phoenix definition was acceptable (5-year: 92.8%). The 5- year BCR-free rate using the strict definition (PSA ≥ 0.2 ng/mL) was 74.1%. Cox’s proportional hazard regression analysis showed that a higher biological effective dose (BED) of ≥180 Gy2 was the only independent variable that could predict BCR (HR: 0.570, 95% C.I.: 0.327-0.994, p = 0.048). Patients with a higher BED (≥180 Gy2) had a significantly higher BCR-free rate than those with a lower BED (<180 Gy2) (5-year BCR-free rate: 80.5% vs. 67.4%).

**Conclusions:**

A higher BED ≥180 Gy2 promises a favorable BCR-free rate, even if the strict definition is adopted.

## Background

Nowadays, low-dose-rate brachytherapy (LDR-brachytherapy) is one of the curative treatment options for non-metastatic prostate cancer [1-6] alongside radical prostatectomy and intensity modulated radiation therapy (IMRT). Usually, the cut-off value in the definition of biochemical recurrence is prostate specific antigen (PSA) of 0.2 ng/mL for patients who underwent radical prostatectomy [7], and the Phoenix definition (nadir + 2 ng/mL) is used for patients who underwent definitive radiation therapy [8]. In several guidelines [9-11], the oncologic outcome of radiation therapy is considered in the same way as that of radical prostatectomy. However, it is inappropriate to compare the oncologic outcome between surgery and radiation therapy using different definitions (0.2 ng/mL vs. nadir + 2 ng/mL). From a radiation oncologist’s viewpoint, a cut-off value of 0.2 ng/mL is very strict, because the prostate gland itself is still present after radiation therapy. As long as different definitions are used, direct comparison of oncologic outcome between surgery and radiation therapy remains impossible. To address this issue, we evaluated the oncologic outcome in patients who underwent LDR-brachytherapy using both definitions of biochemical recurrence (0.2 ng/mL vs. 2 ng/mL).

## Methods

Two hundreds and three patients who were clinically diagnosed with localized prostate cancer (cT1c-2cN0M0) and underwent LDR-brachytherapy between July 2004 and September 2008 were enrolled in this study. The patients’ characteristics are shown in Table 1. The median age, PSA value at diagnosis, and follow-up period were 70.0 years (range: 51–80), 7.4 ng/mL (range: 3.1-32.1), and 72.0 months (range: 2–107), respectively. We evaluated the biochemical recurrence-free rate using the Phoenix definition. We also used the PSA cut-off value of 0.2 ng/mL to evaluate the biochemical recurrence-free rate with the same definition as that used for radical prostatectomy. If the PSA value after seed implantation reached 0.2 ng/mL or more and showed a confirmatory PSA of 0.2 ng/mL or higher, the patient was defined to have biochemical recurrence the first time a PSA increase was noted. If the PSA value did not fall to less than 0.2 ng/mL, the patient was defined to have biochemical recurrence at the date of seed implantation. A pathologist (K.N.), who was expert in prostate cancer diagnosis, centrally reviewed the Gleason score of all biopsy specimens. This study was performed in compliance with the Helsinki Declaration. The institutional review board approved this study, and informed consent was obtained from all patients after explaining the aim and methods of this study.

**Table 1:** Patients’ characteristics stratified by biochemical recurrence using PSA 0.2 ng/mL definition

	**BCR (-) (n = 150)**	**BCR (+) (n = 53)**	**Total (n = 203)**	***p *** **value**
Age (year)				
Median (range)	70.0 (51–80)	69.0 (55–79)	70.0 (51–80)	0.654^§^
PSA at diagnosis (ng/mL)				
Median (range)	7.4 (3.1-32.1)	7.2 (3.7-16.0)	7.4 (3.1-32.1)	0.834^§^
Biopsy gleason score				
6 or less	92	34	126	
7	50	18	68	
8-10	8	1	9	0.576*
Clinical T stage				
T1c	95	29	124	
T2a	44	20	64	
T2b	8	2	10	
T2c	3	2	5	0.563*
Neo-Adjuvant/Adjuvant				
None	104	37	141	
Neo-Ad (+)	38	15	53	
Ad (+)	4	1	5	
Neo-Ad (+), Ad (+)	4	0	4	0.650*
EBRT				
No	102	44	146	
Yes	48	9	57	0.025*

*Chi-square test and ^§^Mann–Whitney *U* test.

### Treatment

Of all 203 patients, 141 patients did not receive neoadjuvant or adjuvant androgen deprivation therapy (ADT), 5 received adjuvant ADT, and 4 received both neoadjuvant and adjuvant ADT. The remaining 53 received only neoadjuvant ADT. One hundred and forty-six underwent only seed implantation and 57 patients received combination treatment including external beam radiation therapy (Table 1).

Risk classification was according to D’Amico’s risk classification [12]. The numbers of low-, intermediate-, and high-risk patients were 93, 92, and 18 patients, respectively (Table 2). Low-risk patients (cT2a, Gleason score 6, and PSA ≤10 ng/mL) and patients (cT2a and PSA ≤10 ng/mL) whose Gleason score of 3 + 4 with the rate of positive biopsy core less than 50% were treated by seed implantation alone. From July 2004 to April 2007, there were 95 patients who were treated with seed implantation at a prescribed dose of 145 Gy, and 51 patients were treated at a prescribed dose of 160 Gy after May 2007. The other patients received combination treatment including external beam radiation therapy (EBRT). The prescribed dose was 110 Gy. The target portion of EBRT was determined one month after seed implantation, and the patients received 45 Gy (in 25 fractions of 1.8 Gy per fraction) using 10 MV photon energy. The clinical target volume included both the whole prostate and one third of the proximal seminal vesicle.

**Table 2:** Patients’ characteristics stratified by biochemical recurrence using PSA 0.2 ng/mL definition

	**BCR (-) (n = 150)**	**BCR (+)(n = 53)**	**Total (n = 203)**	***p *** **value**
D’Amico risk classification				
Low	65	28	93	
Intermediate	70	22	92	
High	15	3	18	0.400*
BED (Gy2)				
Median (range)	183.8 (136.4-244.0)	171.8 (120.3-227.5)	180.6 (120.3-244.0)	0.009^§^
D90 (%)				
Median (range)	110.6 (90.0-136.9)	108.6 (79.8-126.2)	110.3 (79.8-136.9)	0.106^§^
V100 (%)				
Median (range)	94.5 (85.5-99.5)	93.9 (77.8-98.5)	94.3 (77.8-99.5)	0.124^§^
UD30 (%)				
Median (range)	139.2 (99.4-200.3)	140.6 (104.2-197.9)	139.2 (99.4-200.3)	0.785^§^
R100 (mL)				
Median (range)	0.01 (0.0-1.19)	0.04 (0.0-0.51)	0.02 (0.0-1.19)	0.207^§^

*Chi-square test and ^§^Mann–Whitney *U* test, BED: biological effective dose.

Of all patients, 64 patients were treated with a preplanning method and 139 with an intraoperative planning method by modified peripheral loading techniques using Mick’s applicator [13].

### Postdosimetric evaluation

The therapeutic planning and post-implant dosimetric evaluation were performed using Interplant Version 3.3 (CMS, Inc., St. Louis, USA) planning system.

Post-implant CT scanning and post-implant dosimetric studies were performed by one radiation oncologist (A.I.) at 1 month after seed implantation. The dosimetric parameters analyzed in this study were the minimal percentage of the dose received by 90% of the prostate gland (%D90), the percentage prostate volume receiving 100% of the prescribed minimal peripheral dose (V100), and the biological effective dose (BED). BED was calculated to evaluate an independent factor that can predict PSA bounce, and an α/β ratio of 2 was used [14].

### Statistic analysis

The biochemical recurrence-free rate was estimated by the Kaplan-Meier method. A log-rank test was used for between-group comparison. The statistical difference between the recurrence group and the non-recurrence group for categorical variables was tested by the chi-square test, while that for continuous variables was tested by the Mann–Whitney *U* test. To evaluate an independent variable to predict biochemical recurrence, Cox’s proportional hazard regression analysis was carried out.

All statistical analyses were performed using PASW Statistics 17.0 (SPSS Inc., Chicago, IL, USA). All *p* values of less than 0.05 were considered statistically significant.

## Results

**Figure 1:**
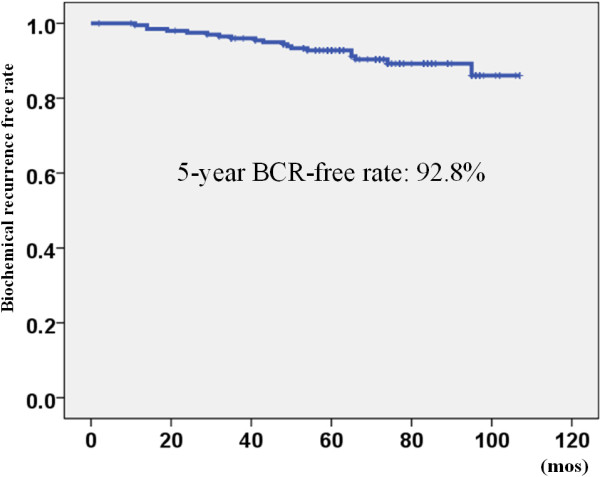
The biochemical recurrence-free rate using the Phoenix definition BCR: biochemical recurrence.

**Figure 2:**
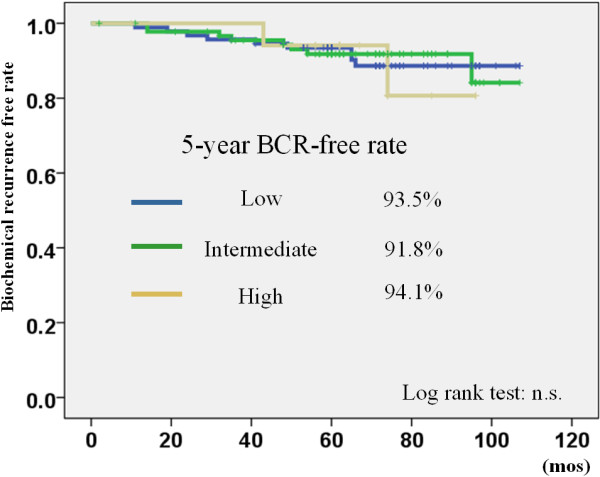
The biochemical recurrence free rate stratified by D’Amico risk classification using Phoenix definition BCR: biochemical recurrence.

Of all patients, 19 patients showed biochemical recurrence according to the Phoenix definition during the follow-up period. The 5-year biochemical recurrence-free rate was 92.8% (Figure 1). The 5-year biochemical recurrence-free rate in low, intermediate, and high-risk patients was 93.5%, 91.8%, and 94.1%, respectively. There was no significant difference between the different risk groups (Figure 2). Regarding clinicopathological parameters (e.g. PSA, Gleason score, age, clinical T stage, BED, D90 (%), and V100), there were no significant differences between patients who showed biochemical recurrence and those who did not show recurrence.

**Figure 3:**
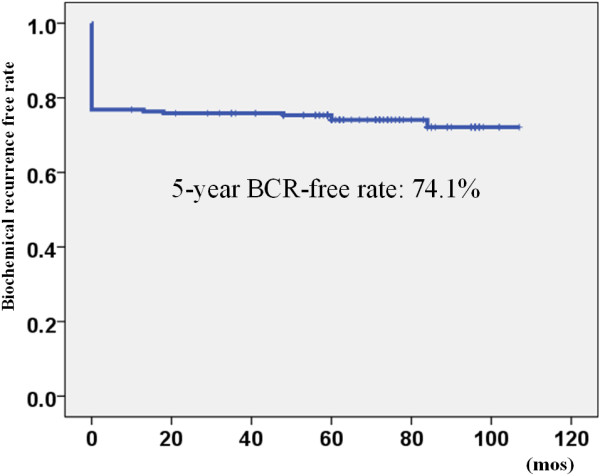
The biochemical recurrence-free rate using the definition of PSA ≥0.2 ng/mL BCR: biochemical recurrence.

**Figure 4:**
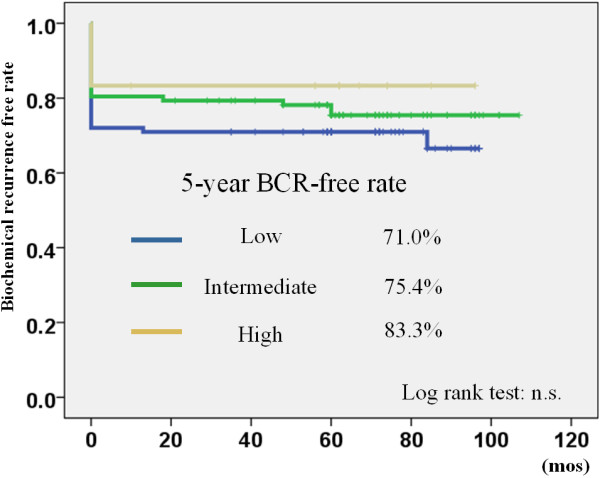
The biochemical recurrence free rate stratified by D’Amico risk classification using PSA 0.2 ng/mL definition BCR: biochemical recurrence.

On the other hand, 53 patients showed biochemical recurrence during the follow-up period according to the definition of PSA ≥0.2 ng/mL. The 5-year biochemical recurrence-free rate was 74.1% (Figure 3). The 5-year biochemical recurrence-free rate in the low, intermediate, and high-risk patients was 71.0%, 75.4%, and 83.3%, respectively. There was no significant difference between the different risk groups (Figure 4). Among clinicopathological parameters, BED in the non-recurrence group was significantly higher than that in the recurrence group (p = 0.009). In all 53 patients who showed biochemical recurrence according to the definition of PSA ≥0.2 ng/mL, 19 (35.8%) met the Phoenix definition, 4 (7.5%) had a PSA value ≥ 1.0 ng/mL, 11 (20.8%) had a PSA value between 0.5 and 0.9 ng/mL, and 19 (35.8%) had a PSA value between 0.2 and 0.5 ng/mL, respectively (Table 3).

**Table 3:** The number of patients and proportion stratified by PSA value of the last follow-up

**PSA (ng/mL)**	**No of pts**	**(%)**
< 0.2	150	73.9
0.2 - 0.49	19	9.4
0.5 - 0.99	11	5.4
1.0 -	4	2.0
Nadir +2	19	9.4

**Figure 5:**
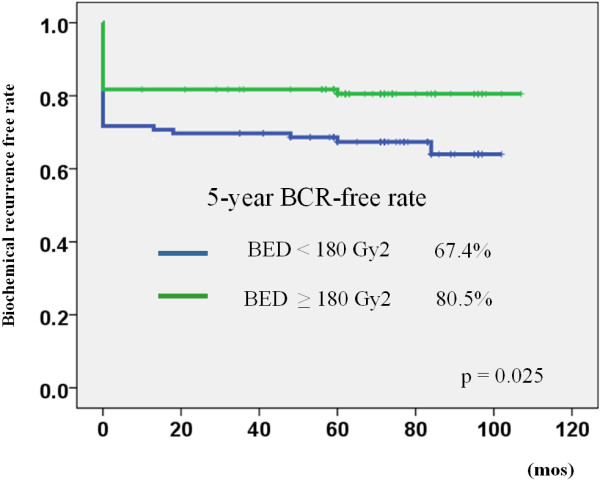
The biochemical recurrence-free rate stratified by biologically effective dose (BED) using the definition of PSA ≥ 0.2 ng/mL BCR: biochemical recurrence.

Cox’s proportional hazard regression analysis showed that a higher BED of ≥180 Gy2 (median BED of all patients) was the only independent variable that could predict biochemical recurrence after seed implantation (HR: 0.570, 95% C.I.: 0.327-0.994, p = 0.048). Patients with a higher BED (≥180 Gy2) had a significantly lower biochemical recurrence rate than those with a lower BED (<180 Gy2) (5-year biochemical recurrence-free rate: 80.5% vs. 68.4%, p = 0.025) (Figure 5).

## Discussion

LDR-brachytherapy has been one of the definitive treatment modalities for prostate cancer alongside radical prostatectomy and IMRT, not only for low-risk patients but also for intermediate and high-risk patients in recent years [11]. The most recent report revealed that the biochemical recurrence-free rate of patients who received LDR-brachytherapy was similar to that of patients who received radical prostatectomy [1-5]. The present study also showed a favorable oncologic outcome according to the Phoenix definition. However, using different definitions to compare the oncologic outcome of radiation therapy to that of radical prostatectomy is questionable. To address this issue, Critz et al. reported the oncologic outcome in patients who received LDR-brachytherapy in combination with EBRT using the same definition for biochemical recurrence as that for surgery [15]. They concluded that the biochemical recurrence-free rate was similar to that of radical prostatectomy using a large cohort study.

The present study shows that the 5-year biochemical recurrence-free rate was 74.1% with a median follow-up period of 72 months. Most patients were classified as low- or intermediate-risk in this cohort. This result gives an unfavorable impression of seed implantation compared to radical prostatectomy. On careful consideration, however, the definition of PSA ≥0.2 ng/mL is very strict for radiation therapy, because the prostate gland itself remains after radiation therapy, while the prostate gland is essentially removed after radical prostatectomy. The remaining prostate gland secretes subtle PSA after radiation therapy. We also defined patients whose PSA did not decrease to less than 0.2 ng/mL as biochemical recurrence in this study. This definition is stricter than that of the Critz study [15]. They defined an increase in 3 consecutive PSA measurements as biochemical recurrence in patients with a follow-up period of less than 5 years. Indeed, 19 patients (9.4%) in our study were defined as biochemical recurrence, but the PSA value at the last follow-up was between 0.2 and 0.49 ng/mL. Taken together, 83.3% of patients showed a PSA value of less than 0.5 ng/mL at the last follow-up (Table 3).

Interestingly, Cox’s proportional regression analysis showed that BED was the only independent variable that can predict biochemical recurrence in this study. We divided all patients by using a 180 Gy2 cut-off value of BED. This was the median value of BED for all patients. The Kaplan-Meier curve showed a significant difference in the 5-year biochemical recurrence-free rate (Figure 5: 80.5% vs. 67.4%, p = 0.025 by the log rank test). This result indicated that a higher BED achieved a higher biochemical recurrence-free rate using PSA ≥0.2 ng/mL definition. Stone et al. also showed that BED was an independent variable that can predict the cancer-free rate of biopsy after seed implantation [16].

To compare the oncologic outcome in the same category such as IMRT vs. brachytherapy, it is appropriate to use the Phoenix definition. However, it is difficult to compare the oncologic outcome between different modalities (e.g. surgery vs. radiation therapy) using different definitions. Nielsen et al. reported that the biochemical recurrence-free rate was overestimated by the Phoenix definition compared to the standard definition (PSA ≥0.2 ng/mL) in patients who underwent radical prostatectomy [17]. They insisted that the 5-year biochemical control rates with a definition of 0.2 ng/mL or higher should be compared with the 10-year biochemical control rates using the Phoenix definition.

In the present study, we demonstrated that most patients (83.3%) achieved a PSA level of less than 0.5 ng/mL with a median follow-up period of 72 months after seed implantation. Patients who achieved a higher BED ≥180 Gy2 also showed a favorable biochemical recurrence-free rate (80.5%) using the definition of PSA ≥ 0.2 ng/mL (Figure 5). We mostly treated the patients in the present study cohort by preplanned methods and the prescribed dose was 145 Gy for monotherapy (95/146: monotherapy pateints). We have now escalated the prescribed dose to 160 Gy and have adopted a real-time planning method. The BED of current patients is at least 180 Gy2. We believe that the oncologic outcomes of the current patient series will be more favorable than those in the present study. We should compare the oncologic outcomes of radical prostatectomy and LDR-brachytherapy using the same definition.

The limitation of this study is the small number of patients. We did not discuss an inter-risk group comparison due to the small number of patients, especially in the high-risk group (n = 18). Indeed, the oncologic outcomes of high-risk group were not different compared with other risk groups (low and intermediate) by either definition. Longer follow-up periods will allow further evaluation to make a definitive conclusion.

## Conclusion

We reported the oncologic outcomes in Japanese patients who underwent LDR-brachytherapy with a median follow-up period of 72 months using different definitions of biochemical recurrence. Higher BED can promise a favorable biochemical recurrence-free rate, even if a strict definition is adopted.

## Abbreviations

BCR: Biochemical recurrence; LDR-brachytherapy: Low-dose-rate brachytherapy; BED: Biological effective dose; PSA: Prostate specific antigen; ADT: Androgen deprivation therapy; EBRT: External beam radiation therapy; % D90: The minimal percentage of the dose received by 90% of the prostate gland; V100: The percentage prostate volume receiving 100% of the prescribed minimal peripheral dose.

## Competing interests

The authors declare that they have no conflicts of interest.

## Authors’ contributions

TN, FK, and HM conceived of this study. AI, KM, and NT participated in data collection. FK and HA helped to draft the manuscript. TN carried out the statistical analysis. All authors read and approved the final manuscript.
